# Preparation of caffeic acid grafted chitosan self-assembled micelles to enhance oral bioavailability and antibacterial activity of quercetin

**DOI:** 10.3389/fvets.2023.1218025

**Published:** 2023-07-05

**Authors:** Xin Ren, Juan Ren, Yipeng Li, Sikun Yuan, Gengnan Wang

**Affiliations:** ^1^College of Veterinary Medicine, Hebei Agricultural University, Baoding, Hebei, China; ^2^Baoding Institute for Food and Drug Control, Baoding, Hebei, China

**Keywords:** quercetin, chitosan, caffeic acid, white feather broiler, bioavailability

## Abstract

Quercetin (QR) is a naturally occurring flavonoid organic compound that has poor solubility in water and highly unstable in alkaline conditions, resulting in limited absorption in poultry. Consequently, in our experiment, QR was employed as a model compound, encapsulated within the caffeic acid graft chitosan copolymer (CA-g-CS) self-assembled micelles to enhance its solubility, stability and exhibit a synergistic antibacterial effect. The optimization of the formula was carried out using a combination of single-factor experimentation and the response surface method. The in vitro release rate and stability of CA-g-CS-loaded QR micelles (CA-g-CS/QR) in various pH media were studied and the pharmacokinetics in white feather broiler chickens was evaluated *in vivo*. Additionally, the antibacterial activity was investigated using Escherichia coliCMCC44102 and Escherichia coli of chicken origin as the test strain. The results showed the optimized formula for the self-assembled micelles were 4 mL water, 0.02 mg/mL graft copolymer, and 1 mg QR, stirring at room temperature. The encapsulation efficiency was 72.09%. The resulting CA-g-CS/QR was uniform in size with an average diameter of 375.6 ± 5.9 nm. The release pattern was consistent with the Ritger-Peppas model. CA-g-CS/QR also significantly improved the stability of QR in alkaline condition. The relative bioavailability of CA-g-CS/QR was found to be 1.67-fold that of the reference drug, indicating a substantial increase in the absorption of QR in the broiler. Compared to the original drug, the antibacterial activity of CA-g-CS/QR was significantly enhanced, as evidenced by a reduction of half in the MIC and MBC values. These results suggest that CA-g-CS/QR improves the bioavailability and antibacterial activity of QR, making it a promising candidate for clinical use.

## 1. Introduction

The excessive use of antibacterial drugs in the livestock and poultry industry has caused significant harm to the health of both animals and humans, such as drug poisoning and antibiotic resistance (1). As people's living standards improve and modern farming practices become more widespread, there is growing concern about the safety of these drugs in the food supply. As a result, some antibacterial drugs used for growth promotion in livestock and poultry are being banned or restricted. With advances in pharmacological technology, it has been shown that many natural products are safe and have various beneficial effects. Quercetin (QR), a representative molecule in the flavonoids family, is being increasingly studied as a potential alternative to antibacterial drugs in the livestock and poultry industry (2, 3).

QR, commonly known as oak essence, is a naturally occurring polyhydroxy flavonoid found in fruits, vegetables, and other natural plants, it exhibits various pharmacological activities, such as an antioxidant (4), anti-inflammatory (5), anti-cancer (6), and antibacterial (7) effects. Moreover it has a positive impact on livestock and poultry, reproduction (8), egg-laying performance (9), immunity (10), etc. Despite its structure containing five phenolic hydroxyl groups, its molecular crystals overlap closely, causing its solubility in water to be limited, only 0.17–7.70 μg/mL (11). This affects its *in vivo* absorption and biological activity, especially in alkaline aqueous solutions, which may destroy QR's structure by hydroxyl ions. Studies have shown that its solubility in water, simulated gastric and simulated intestinal fluids are 7.7 μg/ml, 5.5 μg/ml, and 28.9 μg/ml, respectively. Its oral bioavailability in rats is <17% and even lower in humans, making its use in traditional dosage forms limited (12, 13).

Efforts have been made to overcome these challenges, by incorporating QR into various carriers, such as polymeric conjugates (14), micelles (15), emulsions (16), liposomes (17) and nanocrystals (18). The modification of QR nanoparticles with silver nanoparticles showed a significant improvement in antibacterial effect against drug-resistant Escherichia coli and Staphylococcus aureus (19). Compared to pure QR, the nanoemulsions formulated with QR not only resolved immiscibility and unfavorable interactions, but also displayed greater antioxidant and antibacterial activity and were more effective against Gram-negative bacteria (20). In general, the utilization of natural polymers as drug carriers offers advantages, including low toxicity, versatility in modifying surface properties, increased water solubility, superior biocompatibility, protection of the encapsulated drug, and improvement in stability and prolonged plasma half-life (21, 22).

Chitosan (CS) is a unique cationic polysaccharide with properties such as mucoadhesion, adhesion, film formation, and metal chelation (23). Previous research conducted by our team has demonstrated that chitosan significantly enhances oral absorption. This may be attributed to the reduction in tight junction integrity and improved intestinal absorption facilitated by chitosan (24). However, its limited solubility in water restricts its application. Chemical modification can enhance its solubility and impart new properties to CS (25). Among the various chemical modification methods, the graft copolymerization reaction is the most widely used (26). Additionally, phenolic acids have been effectively covalently linked to CS through graft copolymerization reactions, leading to improve the solubility and biological activity of CS (27, 28).

Caffeic acid (CA) is a phenolic acid compound that exhibits widespread distribution in plants and possesses numerous biological activities. It is characterized by a relatively simple chemical structure and rapid metabolism. Given its status as a widely distributed secondary metabolite in plants, there has been a growing interest in investigating the synthetic pathways and conformational relationships of CA and its derivatives in recent years (29). Extensive research has demonstrated the natural fungicidal properties of CA, which effectively inhibits a diverse range of fungi and bacteria. The inhibitory impact of caffeic acid on E. coli stems from its ability to disrupt the cell wall and membrane structure of bacteria, thereby enhancing cell permeability and causing the leakage of cellular contents. This disruption ultimately leads to the demise of the bacterial cells. (30, 31). The application of CA in derivative design for the synthesis of novel physiologically active green compounds holds considerable practical significance.

Hence, the purpose of this study was to assess the viability of graft copolymer of chitosan and caffeic acid loaded with quercetin micelles (CA-g-CS/QR) in enhancing the stability and solubility of QR and to examine its antibacterial properties and pharmacokinetics in animals following instillation administration, this aims to expand the clinical application of QR.

## 2. Materials and methods

### 2.1. Materials and reagents

QR (≥97%), kaempferol (≥98%), caffeic acid (CA, ≥98%), chitosan (CS, 90% deacetylation), all the above reagents were purchased from Source Leaf Bio (Shanghai, China). DMSO was purchased from Comio Chemical Reagent Co., Ltd. (Tianjin, China). Phosphoric acid was purchased from Beichen Founder Reagent Factory (Tianjin, China). Chromatography grade methanol purchased from Fisher Chemical (USA). The entire experiment utilized ultrapure water.

### 2.2. Experiment animals

The experimental procedures were reviewed and approved by the Institutional Animal Care and Use Committee of Hebei Agricultural University and carried out in accordance with the Guidelines for the Care and Use of Laboratory Animals of China. In this study, 12 healthy 30 days old Chinese white-finned broilers were used for the trial. All broilers were reared from 1 day of age and were provided with adequate basal diets and water, which were formulated to meet or exceed the nutritional requirements of broilers. Room temperature was maintained at 35°C for the first 3 days, then decreased to 28–30°C for the next 2 weeks and 25°C for the rest of the period. Broilers were randomly divided into two treatment groups (six broilers per treatment, *n* = 6) before the experiment, fasted for 18 h, and watered ad libitum. The experiment was administered by instillation, and blood was collected under the wings during the experiment according to the time designed for the experiment, and the plasma was centrifuged and separated for storage in a refrigerator at −20°C for processing and analysis. At the end of the experiment, broilers were euthanized.

### 2.3. Establishment of a method for the determination of QR

QR was determined by UV spectrophotometric method by weighing an appropriate amount of QR, dissolving it in methanol and then diluting it to a certain multiple, and scanning it in the wavelength range of 200~600 nm with methanol as the blank control. The results showed that QR had the maximum absorption at 256 nm, so 256 nm was used as the detection wavelength of QR. This assay was developed for the determination of QR content in in vitro assays.

### 2.4. Preparation of CA-g-CS/QR

#### 2.4.1. Preparation of graft copolymers of chitosan and caffeic acid

0.5 g of CS was completely dissolved in 50 mL of 1% acetic acid solution. subsequently, 1.32 g of ascorbic acid and 1 g of caffeic acid were added. the pH of the solution was adjusted to 6.0 and nitrogen was allowed to flow slowly through the reactor for 60 min. thereafter, 0.375 mL of 10 mol/L hydrogen peroxide was added to the reactor to initiate the reaction. The reaction was carried out under a continuous flow of nitrogen for 16 h. The reacted solution was transferred to a dialysis bag and dialyzed in ultrapure water for 72 h. After dialysis, it was transferred to a flat dish and placed in a freeze dryer for drying (32).

#### 2.4.2. Determination of critical micelle concentration of copolymers

The critical micelle concentration (CMC) of chitosan and caffeic acid graft copolymer (CA-g-CS) was determined by fluorescence spectrophotometry with pyrene as the fluorescent probe, the prepared copolymer solution was diluted step by step, 50 μL pyrene/acetone solution (6 × 10^−5^ mol/L) was added to 10 mL different concentration copolymer solutions, vortex for 2 min, sonicated for 2 h and shaken at 37°C without light overnight for putting the probe pyrene into micelles. After the acetone was completely evaporated, cooled to room temperature, and then measured. The measurement was carried out under the condition that the excitation wavelength was 350 nm, and the slit width of both excitation and emission was 5.0 nm. The intensity ratio of the absorption peaks at the first peak (373 nm) and the third peak (383 nm) was selected as the vertical coordinate, and the logarithm of the concentration was used as the horizontal coordinate to make a graph to obtain two straight lines, and the intersection point was CMC.

#### 2.4.3. Preparation of CA-g-CS/QR

A certain concentration of CA-g-CS were prepared according to the above steps, and QR powder was dissolved in a small amount of methanol, and then this QR solution was slowly dropped into the CA-g-CS solution under high-speed stirring at room temperature and stirred overnight to allow the methanol to evaporate fully.

### 2.5. Optimization of preparation process conditions

#### 2.5.1. Single-factor examination of process conditions

Single-factor experiments were conducted with different weights of QR powder (0.5, 1, 2, and 3 mg), different temperature conditions (25, 37, and 60°C) conditions, different concentrations of CA-g-CS (0.01, 0.02, 0.05, and 0.1 mg/mL), and different volumes of CA-g-CS (2, 4, and 6 mL) as the influencing factors, and the solutions obtained from each factor were filtered and subjected to UV detection. Three parallel experiments were performed for each factor, and the formulation encapsulation rate was calculated according to the following equation:


$$\begin{array}{l} \text{Encapsulation rate }\left(\text{\%}\right)={\text{W}}_{\text{package}}/{\text{W}}_{\text{total}}\times \;100\text{\%} \end{array}$$

Where, W_package_ is the amount of encapsulated QR in the micelles and W_total_ is the total amount of QR in the system.

#### 2.5.2. Optimization of preparation conditions by response surface method

According to the results of the single-factor experiments, it is clear that the increase in temperature did not significantly improve the encapsulation rate of CA-g-CS/QR. Therefore, weight of QR, concentration and volume of CA-g-CS, were selected as independent variables to design a 3-factor, 3-level experiment with encapsulation rate as the response value, and the software Design-Expert was used to optimize the process parameters.

### 2.6. Characterization of CA-g-CS/QR

We evaluated the successful preparation of CA-g-CS by UV absorption and IR detection of CS, CA, and CA-g-CS and compared the solubility of CS as well as CA-g-CS in water. The morphology of CA-g-CS/QR and QR was characterized by transmission electron microscopy. CA-g-CS/QR and QR were dropped on clean copper grids, negatively stained with 2% phosphotungstic acid solution, and the water was evaporated in the air at room temperature. The samples were dried and placed under transmission electron microscopy to observe the morphology.

### 2.7. Determination of encapsulation rate

The CA-g-CS/QR solution was prepared according to the above method, filtered, and diluted to a certain concentration by adding methanol for UV detection and calculating the encapsulation rate according to the above encapsulation rate formula. All analyses were performed using three batches of samples.

### 2.8. *In vitro* release performance

According to the pre-experiment to make QR reach the leaky tank condition and stable, 25% DMSO solution was selected as the release medium. The CA-g-CS/QR solution was prepared, and the above solution was aspirated into a dialysis bag, tied at both ends, placed in 100 mL of release medium, and placed in a water bath at 37°C for release evaluation. Sampling times were 0.5, 1, 1.5, 2, 3, 4, 6, 8, and 10 h, with 1 mL of sample and 1 mL of rehydration solution. UV measurements were performed at the end of sampling and the cumulative release was calculated. Three batches of samples were used for all analyses. CA-g-CS/QR and QR were placed in release media with different pH values and a pharmacokinetic model was fitted to evaluate the release behavior based on the release data.

### 2.9. Stability studies

The CA-g-CS/QR solution and QR were subjected to different pH conditions, and samples were taken periodically to determine the retention rate of QR and to investigate the effect of pH value on the stability of QR.

### 2.10. *In vivo* pharmacokinetic studies

#### 2.10.1. Determination of QR

The Waters Acquity UPLC H-Class system (Waters, Milford, MA, USA) includes a four-stage solvent manager, an autosampler set at 4°C and a Waters Acquity UPLC Shield RP18 column (100 × 2.1 mm, 1.7 μm, Waters. Milford, MA, USA). The mobile phase was methanol-0.1% phosphate (72:28, v/v). Detection was performed by a photodiode array detector at 373 nm at a flow rate of 0.2 mL/min. This assay was developed for the determination of QR in blood plasma.

#### 2.10.2. Preparation of calibration standards

The standard stock solutions of QR and kaempferol were prepared in methanol/0.1% phosphoric acid (4:1, v/v). A series of QR working standard solutions at different concentrations (50.0–5000.0 ng/mL) were prepared by diluting the stock solution. 200 μL of blank plasma were combined with 20 μL of the appropriate QR working solution to create calibration standards, which were then used to create a range of standard solutions with concentrations of 5.0, 10.0, 20.0, 50.0, 100.0, 200.0, and 500.0 ng/mL. Three separate levels of quality control samples representing low, medium, and high QR concentrations of 10.0, 100.0, and 500.0 ng/mL in plasma were generated similarly to the aforesaid procedure.

#### 2.10.3. Plasma sample processing

A 200μL aliquot of plasma was sequentially added 20μL of 40μg/mL kaempferol internal standard, 100 μL of 25% HCL and 2 mL of ethyl acetate vortexed for 2 min equally. This mixture was centrifuged at 5000 rpm for 10 min, and the supernatant was transferred into a centrifuge tube and then concentrated to dryness under nitrogen. The analytes were dissolved in 200 μL of methanol/0.1% phosphoric acid water (4:1, v/v).

#### 2.10.4. Linear relation detection

A series of QR standard solutions with different concentrations were prepared by dilution, 200 μL of blank plasma was taken and 20 μL of QR standard solution was added to obtain standard plasma sample solutions in the concentration range of 5.0~500.0 ng/mL, and the linear regression of QR concentration in plasma was performed by plotting the peak area ratio of QR to kaempferol.

#### 2.10.5. Precision and accuracy and stability

Precision and accuracy were assessed by running the standards (*n* = 6) for 1 day and three consecutive days for three levels of concentration (10.0, 100.0, and 500.0 ng/ml) at low, medium, and high. The stability of QR in the samples was also investigated by sampling and analyzing the three levels of spiked plasma at −20°C for 1 week and 4°C for 24 h.

#### 2.10.6. Pharmacokinetic experimental design

White-finned broilers were randomly divided into two experimental groups, one for the QR solution group and the other for the CA-g-CS/QR group, with six replicates in each group. The CA-g-CS/QR solution and the original QR solution were prepared and administered to the chickens at a dose of 36 mg/kg, and blood was collected under the wings at 15, 30, 60, 120, 240, 360, 480, 720, 1440, and 2160 min after the administration, placed in sodium heparin anticoagulation tubes and centrifuged to separate the plasma.

#### 2.10.7. Pharmacokinetic data analysis

Pharmacokinetic parameters were estimated by blood concentration-time curves from Phoenix WinNonlin software. The non-atrial model was used to calculate the terminal elimination half-life (t_1/2_, λ*z*), the area under the blood concentration-time curve (AUC_0 − t_), terminal feed time (AUC_0−∞_), total clearance (CL), and volume of distribution (V_d_, _λ*z*_). The peak blood concentration (C_max_) and time to peak (T_max_) were read directly from the observed individual blood concentration-time data. For instillation administration, the relative bioavailability (F) was calculated using the AUC_0−∞_ of CA-g-CS/QR versus QR.

### 2.11. Antibacterial test

We selected Escherichia coli CMCC44102 and Escherichia coli of chicken origin as experimental strains to determine their Minimum Inhibitory Concentration (MIC) and Minimum Bactericidal Concentration (MBC). The culture medium was configured and autoclaved, and the bacteria were cultured. The bacterial broth of the logarithmic growth period was diluted by micro broth twofold dilution method, 100 μL broth was added to each well of a sterile 96-well plate, and then 100 μL of prepared drug solution was added to the first well respectively, mixed well, and then diluted in multiples until the last well was mixed and 100 μL of the mixture was discarded. Each well was added with 100 μL of the prepared bacterial diluent, and a positive control (with bacteria and no drug) and a negative control (without bacteria and no drug) were set up. 24 h of incubation at 37°C was taken out and bacterial growth was observed, and each group of experiments was repeated at least three times, and the lowest antibacterial drug concentration contained in the wells without bacterial growth was the MIC. The tritriazolium chloride (TTC) method was used to determine the MIC results. 5 g/L TTC was added to each well of the 96-well plate after incubation for 1 h at 37°C, and the MIC was determined as the lowest antibacterial drug concentration in the wells with bacterial growth and the wells without bacterial growth. 100 μL of the liquid from each well clarified in the above test group was spread on solid, and the lowest drug concentration for no colony growth was its MBC. Agar plate experiments were also performed using the Oxford cup method, using CA-g-CS/QR with QR aqueous solution and maintaining the same concentration, sterile water group and CA-g-CS group were set, and three parallel experiments were performed to further confirm the antibacterial activity of the compounds.

## 3. Results

### 3.1. Critical micelle concentration of graft copolymer

**Figure 1:**
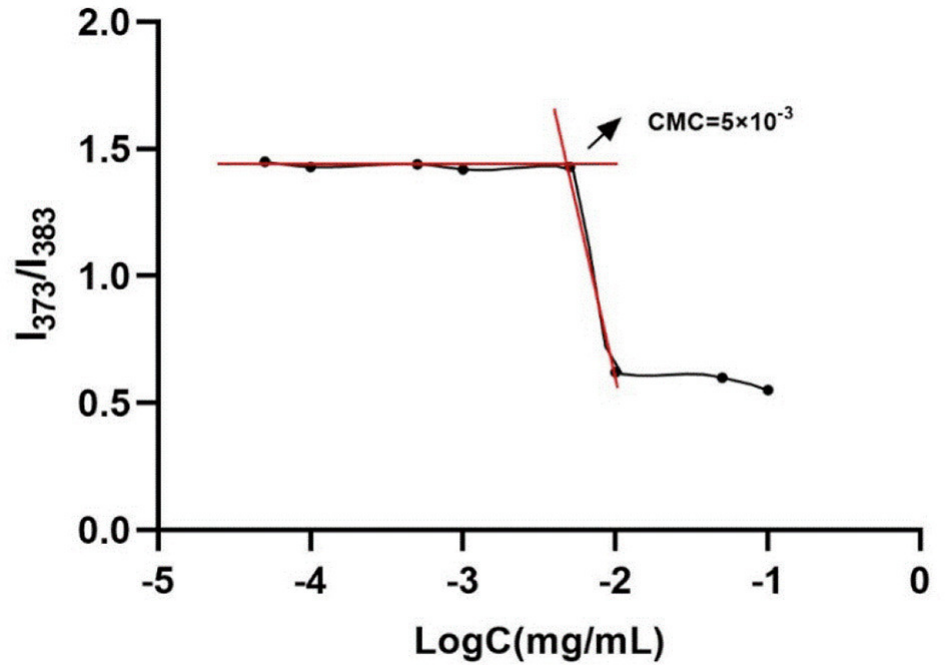
Critical micelle concentration of CA-g-CS(CMC = 5 × 10^−3^).

As shown in Figure 1, the CMC value of CA-g-CS was 5 × 10^−3^ mg/mL, and the results showed that the emission spectrum intensity of pyrene gradually increased with the increase of CA-g-CS concentration. When the CA-g-CS solution concentration was lower than 5 × 10^−3^ mg/mL to make, there was no significant change in the I373/I383 value, and the emission spectrum changed when the CA-g-CS solution concentration was higher than 5 × 10^−3^ mg/mL.

### 3.2. Single-factor experiments

**Figure 2:**
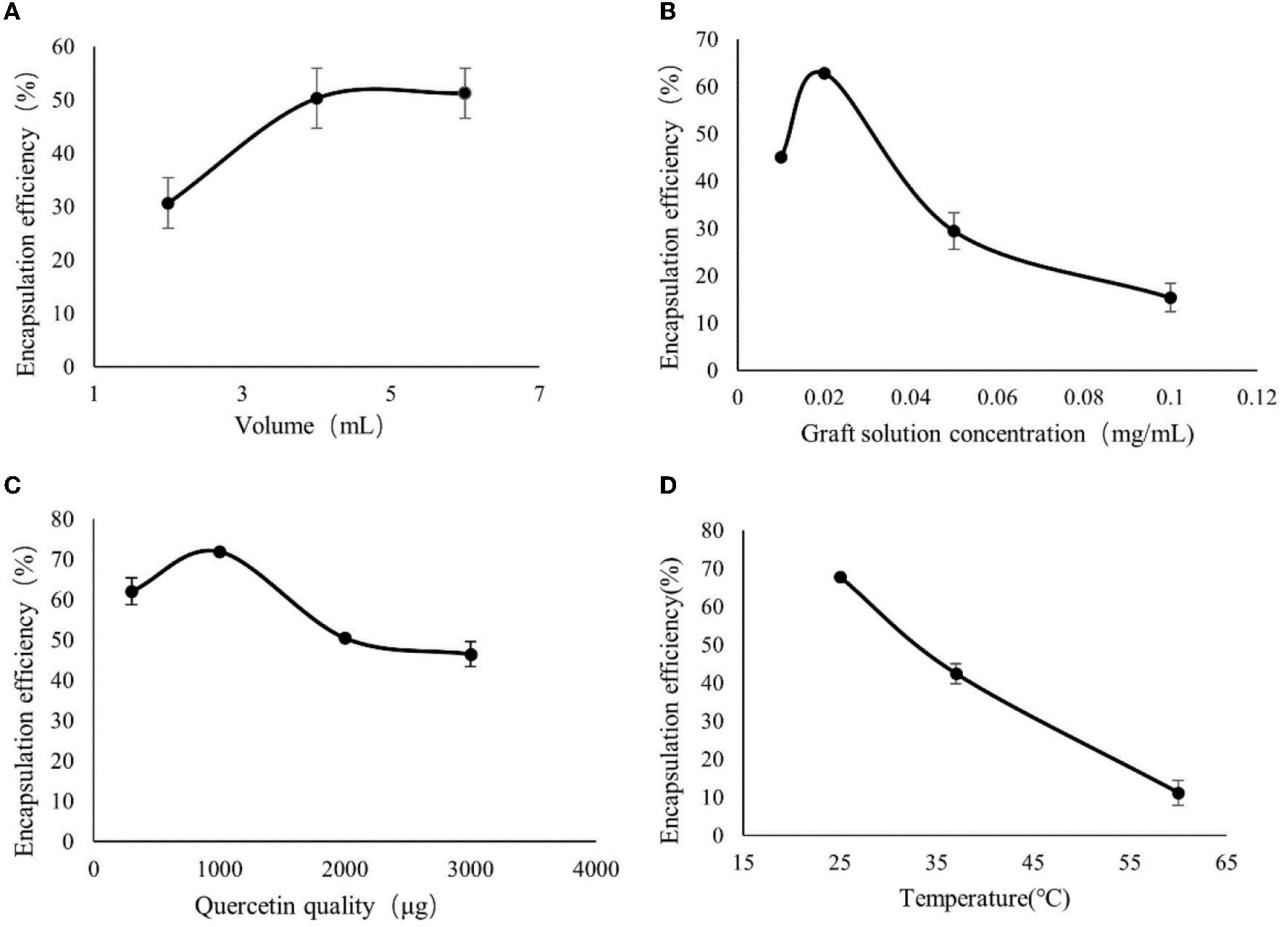
Single-factor experimental results. **(A)** The effect of CA-g-CS solution volume on the preparation of CA-g-CS/QR; **(B)** The effect of CA-g-CS solution concentration on the preparation of CA-g-CS/QR; **(C)** The effect of QR dosage on CA-g-CS/QR preparation; **(D)** The effect of temperature on CA-g-CS/QR preparation.

From Figure 2A, it can be seen that the encapsulation rate of CA-g-CS/QR increased with the increase of the solution volume, and after 4 mL, the effect of volume on the encapsulation rate did not change much, so the best volume was determined to be 4 mL. From Figure 2B, it can be seen that the encapsulation rate decreased instead of increased after the concentration of CA-g-CS reached 0.02 mg/mL, so the concentration of CA-g-CS was chosen to be the best at 0.02 mg/mL. As shown in Figure 2C, the encapsulation rate started to decrease when the amount of QR reached 1 mg, so 1 mg of QR was chosen as the best amount. From the Figure 2D graph, it can be seen that the increase in temperature does not improve the encapsulation rate of CA-g-CS/QR, probably with the increase in temperature, there is a certain effect on the nature of the drug, resulting in a decrease in the encapsulation rate, so the room temperature can be selected for the preparation.

### 3.3. Response surface data

**Figure 3:**
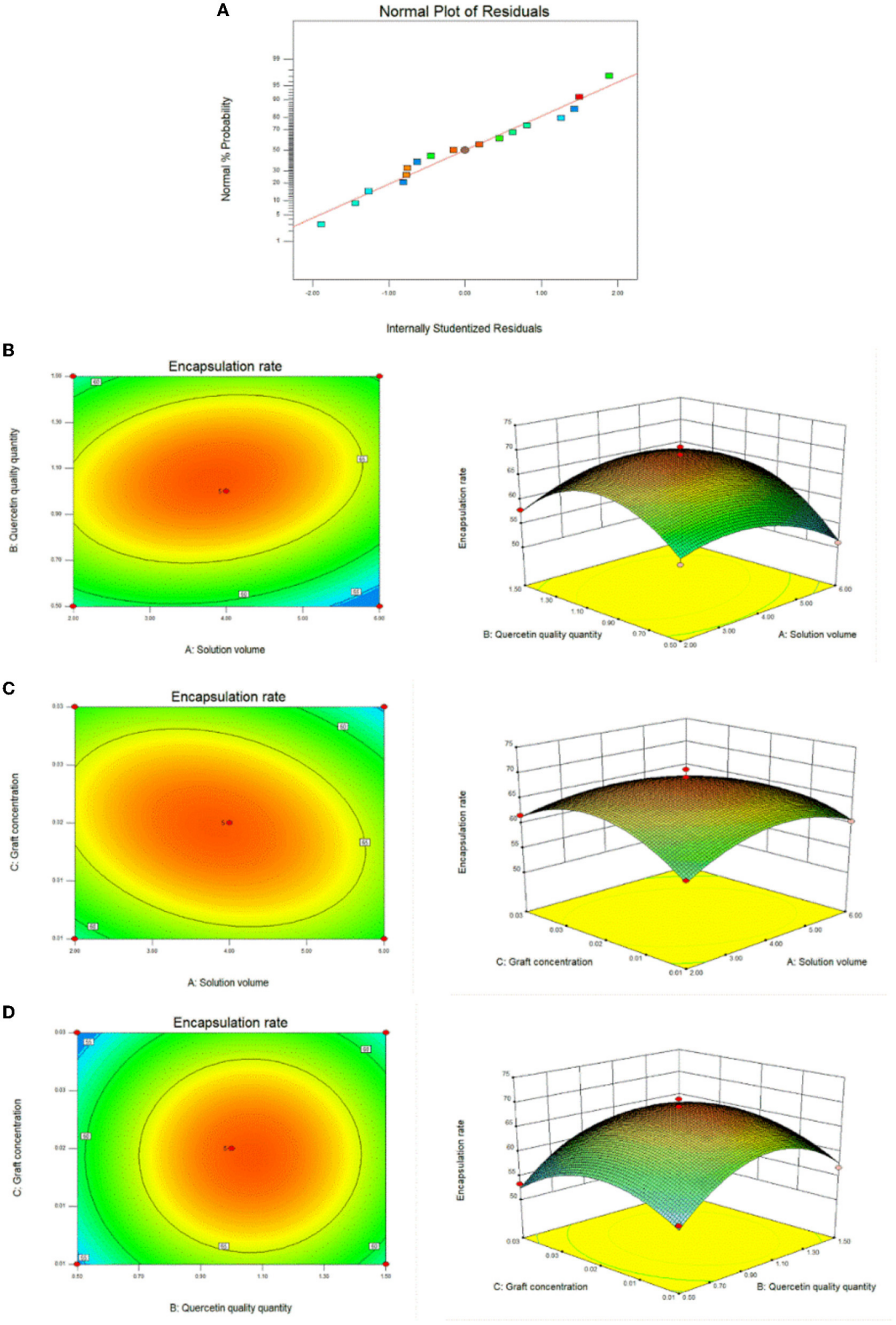
Response surface plots of the interaction effects of different reaction conditions. **(A)** Response surface method to optimize the residual normal plots of the preparation method; **(B)** Response surface plot of the effect of the interaction between solution volume and quercetin amount on the preparation of CA-g-CS/QR; **(C)** Response surface plot of the effect of the interaction of solution volume and graft copolymer concentration on the preparation of CA-g-CS/QR; **(D)** Response surface plots of the effect of the interaction between quercetin amount and graft concentration on CA-g-CS/QR preparation.

We used the results of the single-factor test to optimize the optimal conditions for the synthesis of CA-g-CS/QR. We found that temperature had minimal impact on the results, and the preparation method was improved through the use of the response surface method. The effect of the preparation method variables (A, B, and C) on the dependent variable (Y) was systematically optimized using response surfaces. The regression equation for each factor and response value after regression fitting was Y=69.00-1.01A+1.92B-0.74C+2.09AB-2.48AC+0.062BC-4.49A^2^-7.98B^2^-5.92C^2^. The results showed (Table 1) that the model P < 0.05, indicating that the model was significant. The misfit term = 0.2517 > 0.05 and the model misfit term is not significant. The correlation coefficient R^2^ = 98.11%, indicating that 98.11% of the data could be explained by the equation, which has a high correlation, and the experimental error of the model is reasonable. And the correlation off correction coefficient ${\text{R}}_{\text{Adj}}^{2}$ = 95.67%, indicating that high accuracy and reliability of the experiment (33). The results showed that the influence of each factor on the encapsulation rate was B>A>C in order, and the test points of the normal distribution of residuals (Figure 3A) were basically on the same straight line, indicating that the model could better reflect the true relationship between the factors and the response values, confirming that there was a good correlation between the experimental values. The optimal preparation conditions obtained from the analysis were QR quality amount of 1 mg, solution volume of 4 mL, and graft solution concentration of 0.02 mg/mL. And the response surface analysis plots were obtained based on the regression equation, as shown in Figure 3B, the response surface slope is steeper, indicating that the interaction between the amount of QR and the volume of the solution has a strong effect on the encapsulation rate. While the experimental factors interacting in Figures 3C, D have relatively minor impact on the encapsulation rate.

**Table 1:** Variance analysis of response surface regression model.

**Source**	**Sum of Squares**	**df**	**Mean Square**	**F Value**	***p*-value Prob>F**	
Model	639.24	9	71.03	40.28	<0.0001	Significant
A-Solution volume	8.16	1	8.16	4.63	0.0685	
B-QR quality quantity	29.41	1	29.41	16.68	0.0047	
C-Graft concentration	4.38	1	4.38	2.48	0.1590	
AB	17.43	1	17.43	9.88	0.0163	
AC	24.55	1	24.55	13.92	0.0073	
BC	0.016	1	0.016	8.80	0.9276	
A^2^	84.78	1	84.78	48.08	0.0002	
B^2^	268.28	1	268.28	152.13	<0.0001	
C^2^	147.68	1	147.68	83.74	<0.0001	
Residual	12.34	7	1.76			
Lack of Fit	7.46	3	2.49	2.03	0.2517	Not significant
Pure Error	4.89	4	1.22			
Cor Total	651.59	16				

### 3.4. Validation tests

Based on the results of response surface analysis, the preparation conditions of CA-g-CS/QR were determined as follows: QR 1 mg, CA-g-CS volume 4 mL, and CA-g-CS concentration 0.02 mg/mL. The encapsulation rate under these conditions can reach 70.78%, and the encapsulation rate obtained from the validation test according to this preparation condition is 71.98%, which is very close to the predicted value of the model, indicating that the final optimization of the response surface model The final optimization result of the response surface model is reliable.

### 3.5. Characterization of CA-g-CS/QR.

Supplementary Figures 1, 2 present the UV and IR spectra of CS, CA, and CA-g-CS, respectively. It is noteworthy that CS exhibits limited solubility in water, whereas the CA-g-CS compound synthesized in our study demonstrates favorable solubility in aqueous solutions. The UV spectrograms obtained for CS, CA, and CA-g-CS reveal notable distinctions in the UV absorption spectra among these three compounds. In IR spectra, it can be judged that the new absorption peak of CA-g-CS at 1619.55 cm^−1^ may be caused by the stretching vibration of caffeic acid C=C, which proves that caffeic acid can be successfully grafted onto chitosan. Both UV and IR results verified the successful preparation of CA-g-CS.

**Figure 4:**
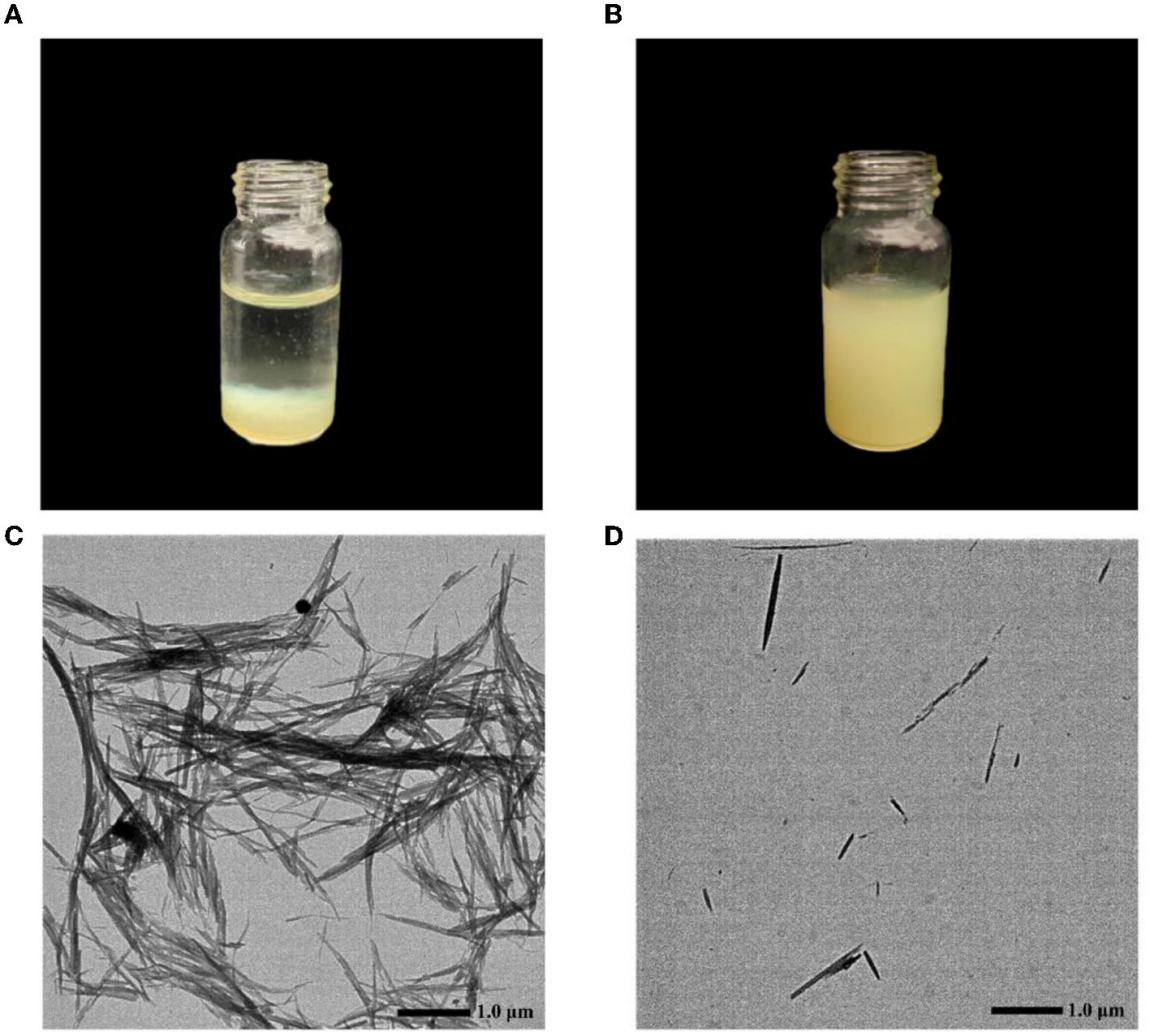
QR and CA-g-CS/QR characterization chart. **(A)** Aqueous QR solution; **(B)** CA-g-CS/QR solution; **(C)** QR transmission electron micrograph, particle size 1.5 ± 0.1μm; **(D)** CA-g-CS/QR transmission electron micrograph, particle size 375.6 ± 5.6nm.

Figure 4A shows aqueous QR solution, and Figure 4B shows CA-g-CS/QR solution, from which it can be observed that aqueous QR solution exhibits poor solubility and rapid sedimentation, while QR solubility increases in the CA-g-CS. The electron micrographs Figure 4D showed that the particle size of the CA-g-CS/QR prepared in this experiment was 375.6 ± 5.9 nm and the CA-g-CS/QR morphology was small, while the QR Figure 4C was not uniform in size and showed larger rod-shaped crystals. The prepared micelles showed uniform dispersion without aggregation and a smaller particle size compared to the original drug, which was characterized by larger rod-shaped crystals.

### 3.6. CA-g-CS/QR encapsulation rate measurement

The encapsulation rate of CA-g-CS/QR was determined to be 72.09%, which improved the difficulty of poor solubility of QR in water. The determination of the encapsulation rate further validated the feasibility of CA-g-CS/QR preparation.

### 3.7. *In vitro* dissolution

**Figure 5:**
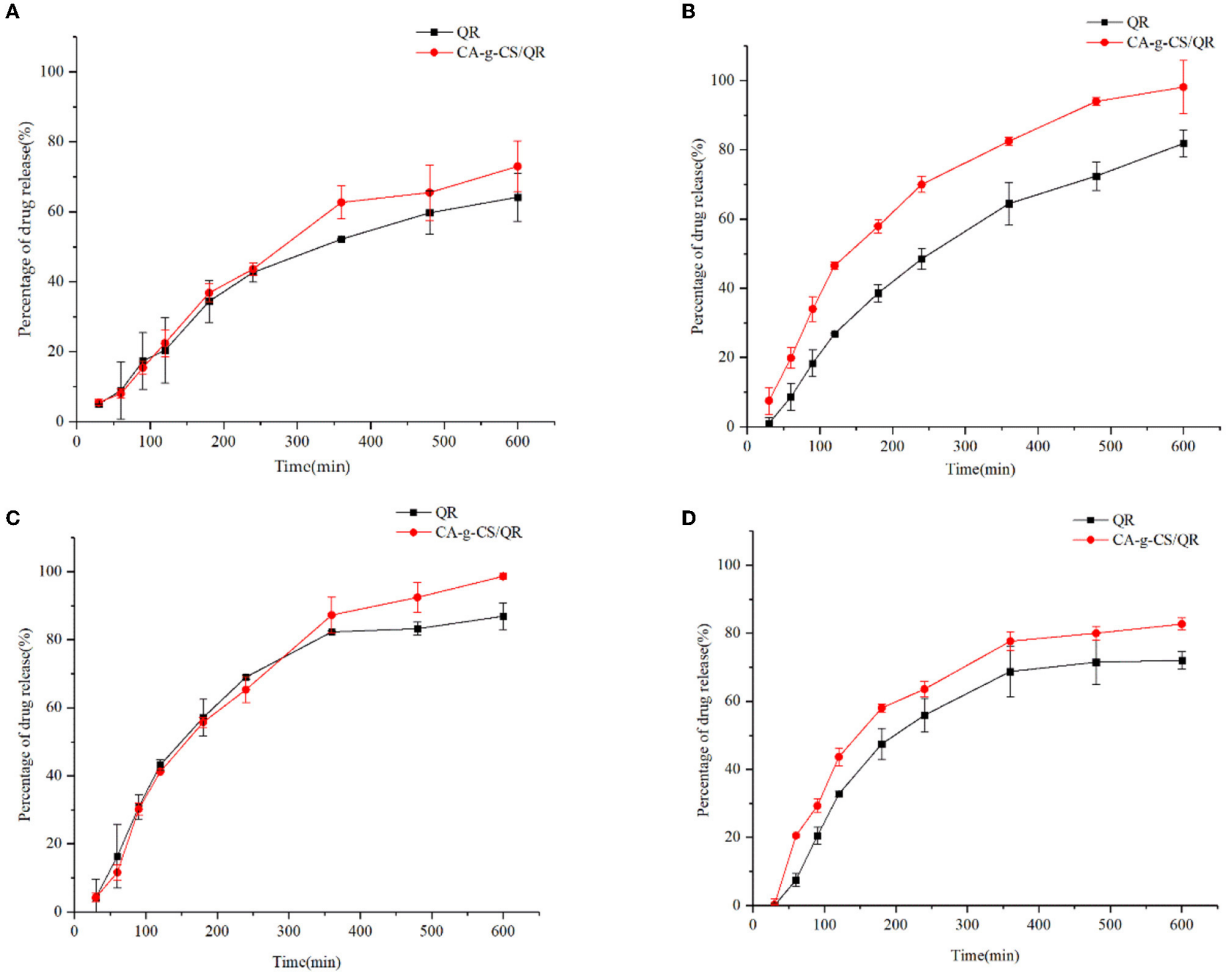
In vitro release analysis of QR and CA in different media. **(A)** Cumulative release of CA-g-CS/QR and QR in 25% DMSO; **(B)** Cumulative release of CA-g-CS/QR with QR in release medium at pH 1.2; **(C)** Cumulative release of CA-g-CS/QR with QR in release medium at pH 6.8; **(D)** Cumulative release of CA-g-CS/QR with QR in release medium at pH 7.4.

*In vitro* release experiments Figure 5A showed that CA-g-CS/QR showed a significant release-promoting effect compared with QR, and the drug release rate reached 75.89%. The drug release rate was faster when the release medium was pH = 1.2 (Figure 5B), and the drug release was relatively stable when the release medium was pH = 6.8 (Figure 5C). CA-g-CS also enhanced the release rate of QR when the release medium was pH = 7.4 (Figure 5D). In order to clarify the release mechanism of CA-g-CS/QR, several mathematical models were used to fit the release kinetics of CA-g-CS/QR, and the fitting results were shown in Table 2. The fitting results were determined according to R^2^. As can be seen from the table, the Ritger-Peppas model has the best regression effect. The Ritger-Peppas model was used to study the mechanism of drug release in different pH media. The R^2^ obtained were 97.72%, 96.72%, and 91.18% respectively, so it is reasonable to choose the Ritger-Peppas model to describe the release mechanism of the drug. The fitted equations were F=3.379t^0.541^, F=2.855t^0.556^, and F=1.667t^0.630^ at pH of 1.2, 6.8, and 7.4, respectively, where the n values were all consistent with 0.45 <n <0.89, so the drug release followed the combined effect of drug diffusion and skeletal dissolution at this time.

**Table 2:** Different kinetic model fitting of CA-g-CS/QR drug release data.

**Function**	**A**	**B**	**C**	**R^2^**
Zero order: y = a·t	0.125 ± 0.013	NA	NA	91.98
First order: y = a·[1- exp(-b·t)]	98.04 ± 11.75	0.0024 ± 0.001	NA	98.01
Higuchi: y = a·t^0.5^	3.90 ± 0.21922	NA	NA	97.53
Ritger-Peppass: y = a·t^b^	0.73 ± 0.33	0.73 ± 0.07	NA	99.04
Weibull: : y = a·[1- exp(-b·t^c^)]	73.52 ± 3.88	0.04 ± 0.001	1.47 ± 0.38	98.35

Data presented as mean ± SD. NA, not available.

### 3.8. Stability test

**Figure 6:**
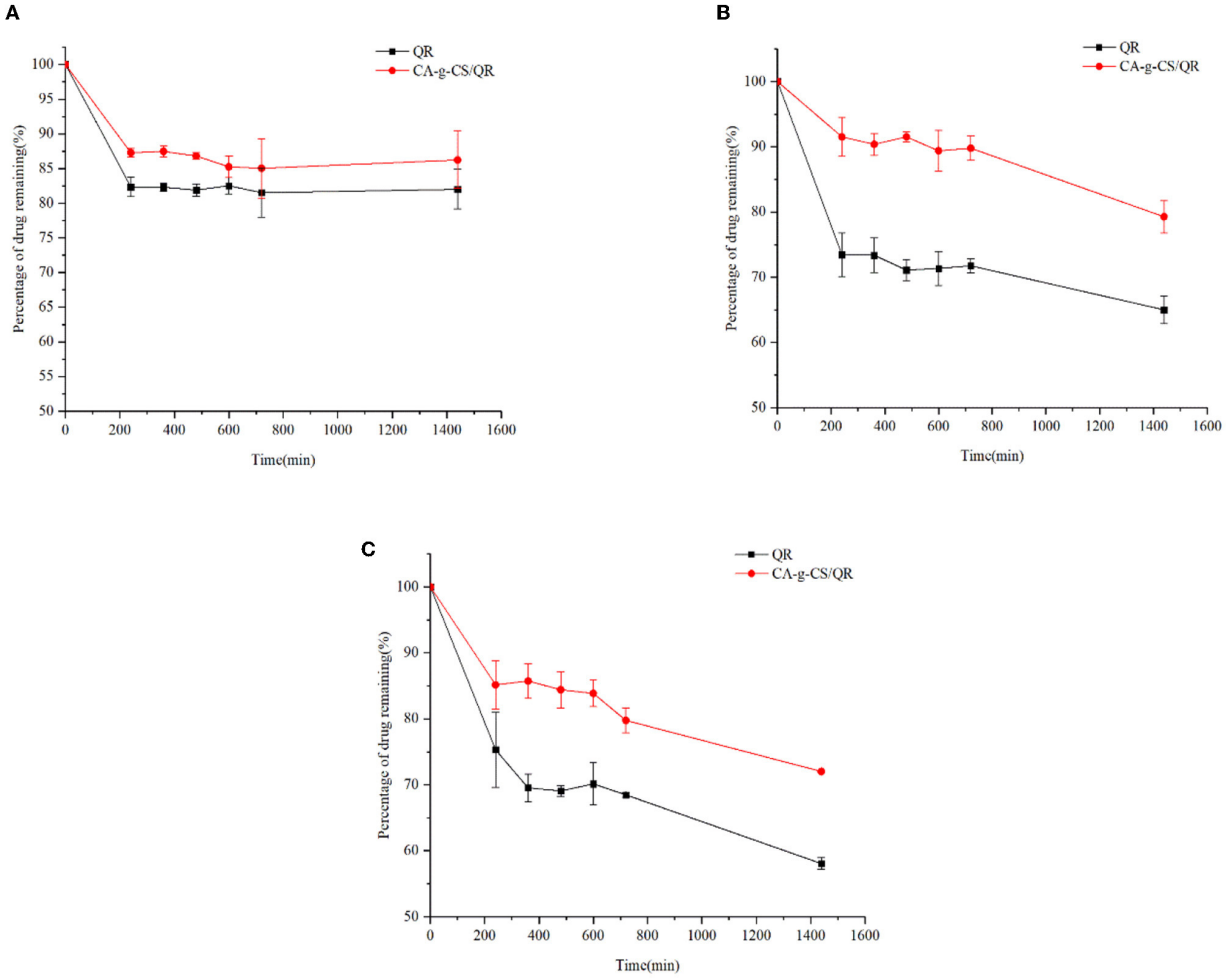
Investigation of QR and CA-g-CS/QR stability in different pH media. **(A)** Investigation of the stability of CA-g-CS/QR and QR in medium at pH 1.2; **(B)** Investigation of the stability of CA-g-CS/QR and QR in medium at pH 6.8; **(C)** Investigation of the stability of CA-g-CS/QR and QR in medium at pH 7.4.

As can be seen from Figure 6, the preservation rate of QR decreased significantly with increasing solution pH, while CA-g-CS/QR was significantly better than QR under different pH environmental conditions. In particular, the preservation rate of CA-g-CS/QR was significantly higher than that of QR solution in the medium with pH 7.4, and the results were consistent with the results of in vitro dissolution experiments.

### 3.9. Pharmacokinetic studies

#### 3.9.1. Methodological validation

The calibration curve is Y = 0.0004x + 0.0087 (20.0–500.0 ng/mL) R^2^ = 0.9993; Y = 0.0004x + 0.0059 (5.0–100.0ng/mL) R^2^ = 0.9993. The data of precision, accuracy, stability, and recovery are shown in Tables 3, 4.

**Table 3:** The within-day and between-day precision, accuracy and stability of this method for the determination of QR in plasma.

**Conc. (ng/mL)**	**Within-run** (***n** =* **6)**		**Between-run** (***n** =* **6, three runs)**		**Recovery (%)**
	**Precision (RSD %)**	**Accuracy (%)**	**Precision (RSD %)**	**Accuracy (%)**	
10	0.15	93.67 ± 0.75	0.07	104.95 ± 8.92	92.18 ± 7.49
100	0.29	97.23 ± 1.46	0.16	101.65 ± 3.47	104.38 ± 6.34
500	0.33	90.34 ± 1.65	0.21	99.63 ± 1.11	99.25 ± 1.29

Each value represents the mean ± SD (n =6).

**Table 4:** Stability of QR in white broiler plasma at −20°C for 7 days and 4°C for 24 h assay.

**Conc. (ng/mL)**	**Remaining (%)**	
	**In plasma stored at** −**20**°**C for 7 days**	**In prepared plasma sample stored at 4**°**C for 24h**
10	96.64 ± 6.62	92.76 ± 2.94
100	103.28 ± 8.97	97.25 ± 5.63
500	93.52 ± 1.78	99.98 ± 0.45

Each value represents the mean ± SD (n = 6).

#### 3.9.2. Pharmacokinetic studies

**Figure 7:**
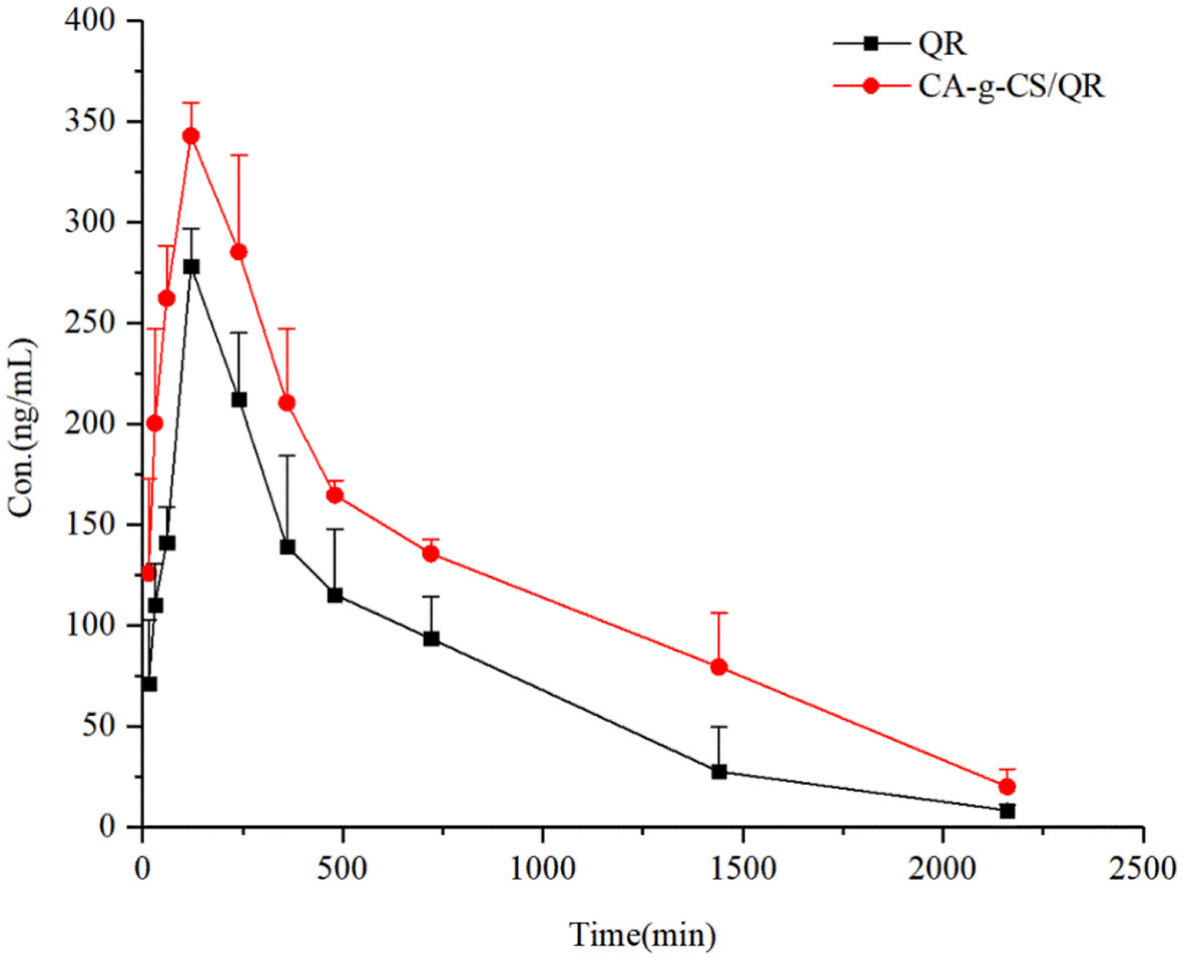
Time profiles of mean blood concentrations in white-finned broiler chickens after infusion of QR or CA-g-CS/QR (i.g., 36 mg/kg) administration (mean ± SD, *n* = 6).

The relationship between the mean blood concentration and time of CA-g-CS/QR and the prodrug is shown in Figure 7, and the pharmacokinetic parameters are shown in Table 5. CA-g-CS/QR increased the absorption of QR in white broiler chickens over 36 h. Compared with the QR prodrug, the C_max_ increased by 1.23 times, the area under the curve AUC_0−∞_ of the CA-g-CS/QR was about 1.7 times that of the prodrug, and the clearance CL decreased to 126.04 mL/min/kg and the bioavailability was 167%, which indicated that the CA-g-CS/QR could promote the absorption of QR.

**Table 5:** Pharmacokinetic parameters after QR or CA-g-CS/QR administration.

**Parameters**	**CA-g-CS/QR**	**QR**
T_max_(min)	120	120
C_max_(ng/ml)	342.96 ± 16.51	278.26 ± 18.52
T_1/2, λz_(min)	556.41 ± 88.97	419.03 ± 42.26
AUC_0 − t_(ng.min/ml)	267757.13 ± 19032.85	165506.99 ± 38159.40
AUC_0−∞_(ng.min/ml)	284773.44 ± 21518.65	170593.89 ± 38072.75
V_d, λz_/F(mL/kg)	101553.0 ± 15421.23	131782.24 ± 25460.13
CL/F (mL/min/kg)	126.04 ± 9.36	219.15 ± 44.09
F (%)	166.93	

Each value represents the mean ± SD (n = 6).

### 3.10. Antibacterial test

**Figure 8:**
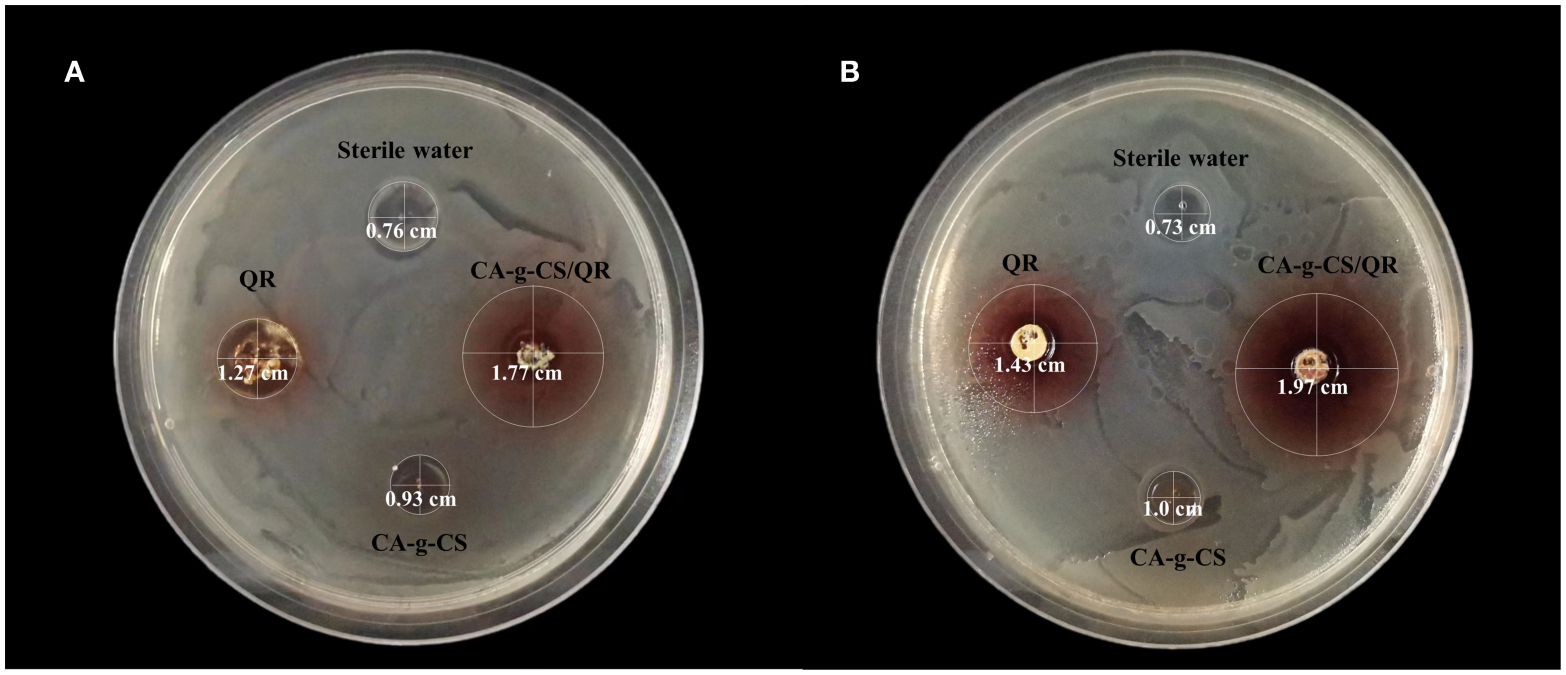
QR and CA-g-CS/QR antibacterial effect chart. **(A)** Effect of QR, CA-g-CS/QR, CA-g-CS aqueous solution and sterile water on the inhibition circle of E. coli CMCC44102 agar plate. The diameter of the inhibition circle is 1.27 cm, 1.77 cm, 0.93 cm, 0.76 cm respectively in order. **(B)** Effect of QR, CA-g-CS/QR, CA-g-CS aqueous solution and sterile water on the inhibition circle of *E. coli* agar plate of chicken origin. The diameter of the inhibition circle is 1.43 cm, 1.97 cm, 1.0 cm, 0.73 cm respectively in order.

CA-g-CS/QR could effectively inhibit the growth of Escherichia coli and chicken Escherichia coli, and the inhibition effect was significant with the increase in CA-g-CS/QR concentration. For Escherichia coliCMCC44102, the measured MIC and MBC of QR were 0.013 mol/L and 0.026 mol/L, while the MIC and MBC of the CA-g-CS/QR were 0.0065 mol/L and 0.013 mol/L. For E. coli of chicken origin, the MIC and MBC measured by QR were 0.0585 mol/L and 0.117 mol/L, respectively, while the MIC and MBC of CA-g-CS/QR were 0.029 mol/L and 0.0585 mol/L, respectively, and the inhibition circle and diameters of each experimental group is marked in Figure 8, and it can be clearly observed that the inhibition activity of CA-g-CS/QR group is better than QR group which judged that the CA-g-CS/QR could significantly improve the antibacterial effect of QR.

## 4. Discussion

The ban on the use of antibiotics as additives in animal production has led to a new consensus in utilizing biologically active chemical chemicals in plants to enhance animal performance and prevent animal illnesses (34). QR exhibits a range of pharmacological properties making it a promising candidate as green feed additives in animal production. Despite its potential benefits, QR's low solubility and bioavailability have prompted research into the synthesis of QR derivatives to improve its properties (2).

To examine the self-assembly behavior of CA-g-CS in water, we employed pyrene as a fluorescent probe. The pyrene interacted with the hydrophobic region of CA-g-CS while its hydrophilic end was exposed to the aqueous environment, leading to the maintenance of micelle stability. The results showed that the emission spectrum intensity of pyrene increased as the concentration of CA-g-CS increased. The polarity of the pyrene's medium affected the intensity of its vibrational peaks in the emission spectrum. When the CA-g-CS solution concentration was below 5 × 10^−3^ mg/mL, the I373/I383 values remained stable, suggesting that CA-g-CS was present as single chains and non-stable micelles. However, when the concentration exceeded 5 × 10^−3^ mg/mL, the copolymer self-assembles in water to form nano micelles, increasing in intermolecular force and transfer of hydrophobic pyrene from the polar water to the non-polar hydrophobic region of the nano micelles, leading to changes in the emission spectra (35).To synthesize CA-g-CS, we employed free radical-mediated graft copolymerization reaction. This reaction method offers several advantages, such as utilizing green materials and reagents, producing a less toxic reaction products, and being economically and environmentally friendly as it can be performed in room temperature and reduces the degradation and oxidation of phenolic acid (36, 37). Self-assembled micelles exhibit exceptional physical and chemical characteristics, allowing for the reduction or avoidance of toxic side effects from drugs on healthy tissues and organs. This is achieved by encapsulating drugs at higher concentrations specifically at the site of the lesion through self-assembly. In comparison to conventional micelles, self-assembled micelles are more straightforward to prepare and widely utilized due to their lack of dependence on emulsifiers and additional media (38, 39).

Chemical composition is the material basis of a drug, so the number and position of absorption peaks on the absorption spectra of the same drug should be the same, and if there are differences in the number, shape and position of absorption peaks, they can be used to differentiate the drugs. We chose UV absorption spectra and IR spectra to verify the successful preparation of CA-g-CS. the solubility of CS in water was poor, while our prepared CA-g-CS showed good solubility in water. the results of UV and IR spectra of CS, CA and CA-g-CS showed significant differences between the three. The successful preparation of CA-g-CS was verified, which laid the foundation for the successful preparation of CA-g-CS/QR. And we used the results of the single-factor test to optimize the optimal conditions for the synthesis of CA-g-CS/QR. We found that temperature had minimal impact on the results, and the preparation method was improved through the use of the response surface method. The model showed a significant results, with a P value of less than 0.05. The model fit was also deemed appropriate as the misfit term (0.2517) was greater than 0.05, and the correlation coefficient (R^2^) was 98.11%, indicating that 98.11% of the data could be explained by the equation. which has a high correlation, and the experimental error of the model is reasonable. Furthermore, the adjusted correlation coefficient (${\text{R}}_{\text{Adj}}^{2}$) was 95.67%, indicating the high accuracy and reliability of the experiment (33). The residuals were found to be normally distributed, further confirming the validity of the model. The optimal preparation condition was determined to be the loading of 1 mg of QR with 4 mL of 0.02 mg/mL CA-g-CS. And the response surface analysis plots were obtained based on the regression equation, and the shape of the response surface was examined after fitting. The optimal process parameters were determined through the analysis of response surface contours, and the interaction between factors could be visualized through the examination of 3D surface plots (40, 41). In the response surface plot, the slope of the surface reflects the level of influence of the test factor on the response value. A steeper slope indicates a greater influence of the test factor on the response value (42). Our experimental results indicate that the interaction between the quantity of QR and the volume of the solution has a significant impact on the encapsulation rate. Additionally, the prepared micelles showed uniform dispersion without aggregation and a smaller particle size compared to the original drug, which was characterized by larger rod-shaped crystals. The determination of the encapsulation rate of the prepared micelles further validated the feasibility of CA-g-CS/QR preparation.

QR is insoluble in water and highly unstable in an alkaline conditions, making it challenging to obtain accurate results in the in vitro release assay with a conventional dissolution medium. However, 25% DMSO was selected as the release medium as it satisfied the leaky tank condition and ensured improved release outcomes. The in vitro release experiments demonstrated that CA-g-CS/QR exhibited a significant release-enhancing effect compared to the QR stock solution. As the CA-g-CS/QR are conjugates, the release of the drug occurs in two stages. The first stage involves the disruption of the encapsulated structure, where the chemically linked chains within the molecule are cleaved before the drug is finally released (43). The combination of CS with drugs can produce a triggered release behavior due to the pH sensitivity of chitosan (44). The weak alkalinity of the release medium and the instability of QR in alkaline solutions caused changes in QR properties in the post-release solution measured after 10 hours, which were consistent with the results of examining the stability of CA-g-CS/QR in the medium solution with pH=7.4. The encapsulation of CA-g-CS provided a protective effect on QR, which became more significant with an increase in solution pH. This is because QR, a flavonoid, is weakly acidic and can exist stably under acidic conditions, but can easily be converted into organic quinones under alkaline conditions (45). The effect of environmental acidity and alkalinity was mitigated through encapsulation with CA-g-CS. Furthermore, the drug release kinetic model was used to fit the drug release data of the particles under different pH conditions. The values of “n” obtained from fitting the drug release data to the Ritger-Peppas model was all consistent with 0.45 <n <0.89, indicating that the drug release was a result of a combination of drug diffusion and skeletal dissolution at this time. These results correspond to the in vitro release performance results.

The clinical effects of QR have been of much interest since the 1990s, and designing new dosage forms have become a priority as traditional forms such as granules or tablets are insufficient for clinical use. Research has indicated that QR micelles have a longer retention time in animals, leading to a higher drug utilization (46). The effects of QR on chicken performance, such as improved eggshell strength and thickness (9, 47) and meat quality and lipid metabolism (48, 49) have been widely studied. While the pharmacokinetic metabolism of QR has been extensively studied in rats, there is limited knowledge in poultry (50, 51). The ultra-high-performance liquid chromatography (UPLC) method was used in this study to determine QR concentration in broiler plasma, with kaempferol as the internal standard, is accurate, sensitive, simple, specific, and reproducible. The results of our *in vivo* pharmacokinetic study demonstrate that the CA-g-CS/QR can reduce the *in vivo* clearance of QR and prolong its elimination half-life, effectively improving its bioavailability in vivo and providing a foundation for future *in vivo* studies of QR in poultry.

QR boasts a broad-spectrum of antimicrobial properties and shows more potent antibacterial effects against Gram-negative bacteria than Gram-positive ones. Its ability to damage cell walls, impede protein/nucleic acid synthesis, and act as a metabolic antagonist is opening up new possibilities for addressing the issue of multidrug resistance in clinical settings (7). Escherichia coli, a common pathogenic bacteria causing infections in livestock and poultry, was targeted in this experiment. Results showed that the MIC and MBC of the CA-g-CS/QR were lower than those of the pure drug, implying that our micelles demonstrate an improved level of bacterial inhibition compared to the original drug. Further research will be conducted to more comprehensively verify this idea.

In conclusion, CA-g-CS/QR not only has a protective effect on QR at lower pH value, slowing down its degradation, but also enhances its antibacterial activity. Most importantly, CA-g-CS/QR showed higher bioavailability *in vivo*, indicating a significant improvement in the formulation performance of QR proagents. The successful preparation of CA-g-CS/QR laid a foundation for further study of insoluble drugs and their pharmacokinetics *in vivo*.

## Data availability statement

The original contributions presented in the study are included in the article/Supplementary material, further inquiries can be directed to the corresponding author.

## Ethics statement

The animal study was reviewed and approved by Institutional Animal Care and Use Committee of Hebei Agricultural University.

## Author contributions

Methodology, formal analysis, and writing—original draft preparation: XR and GW. Validation: XR, JR, and YL. Investigation and writing—review and editing: XR, JR, and GW. Supervision: SY and GW. Project administration and funding acquisition: GW. All authors have read and agreed to the published version of the manuscript.
