# Supplementary material for: Preparation of caffeic acid grafted chitosan self-assembled micelles to enhance oral bioavailability and antibacterial activity of quercetin
Source: Front Vet Sci. 2023 Jul 5;10:1218025. doi: 10.3389/fvets.2023.1218025 (PMC10354432; doi:10.3389/fvets.2023.1218025)

**Preparation of caffeic acid grafted chitosan self-assembled micelles to enhance oral bioavailability and antibacterial activity of quercetin**

**1. CS, CA, CA-g-CS UV-Vis spectra.**

1 mg of the sample was dissolved in 1% acetic acid aqueous solution to make the concentration of each sample consistent, and the sample solution was scanned by spectrophotometer in the range of 200-600 nm. the corresponding UV absorption spectra of CS, CA and CA-g-CS are shown in Figure 1, CS has no obvious absorption peak at 200-600 nm, CA has a characteristic UV absorption peak at 304 nm, and CA -g-CS showed the characteristic absorption peak at 322 nm, and there were obvious differences in the UV absorption spectra of the three. The chemical composition is the material basis of drugs, so the number and position of absorption peaks on the UV absorption spectra of the same drug should be the same, if there are differences in the number, shape and position of absorption peaks, they can be used to distinguish the drugs. Therefore, from the results of the figure, it can be concluded that CA-g-CS has been successfully synthesized.


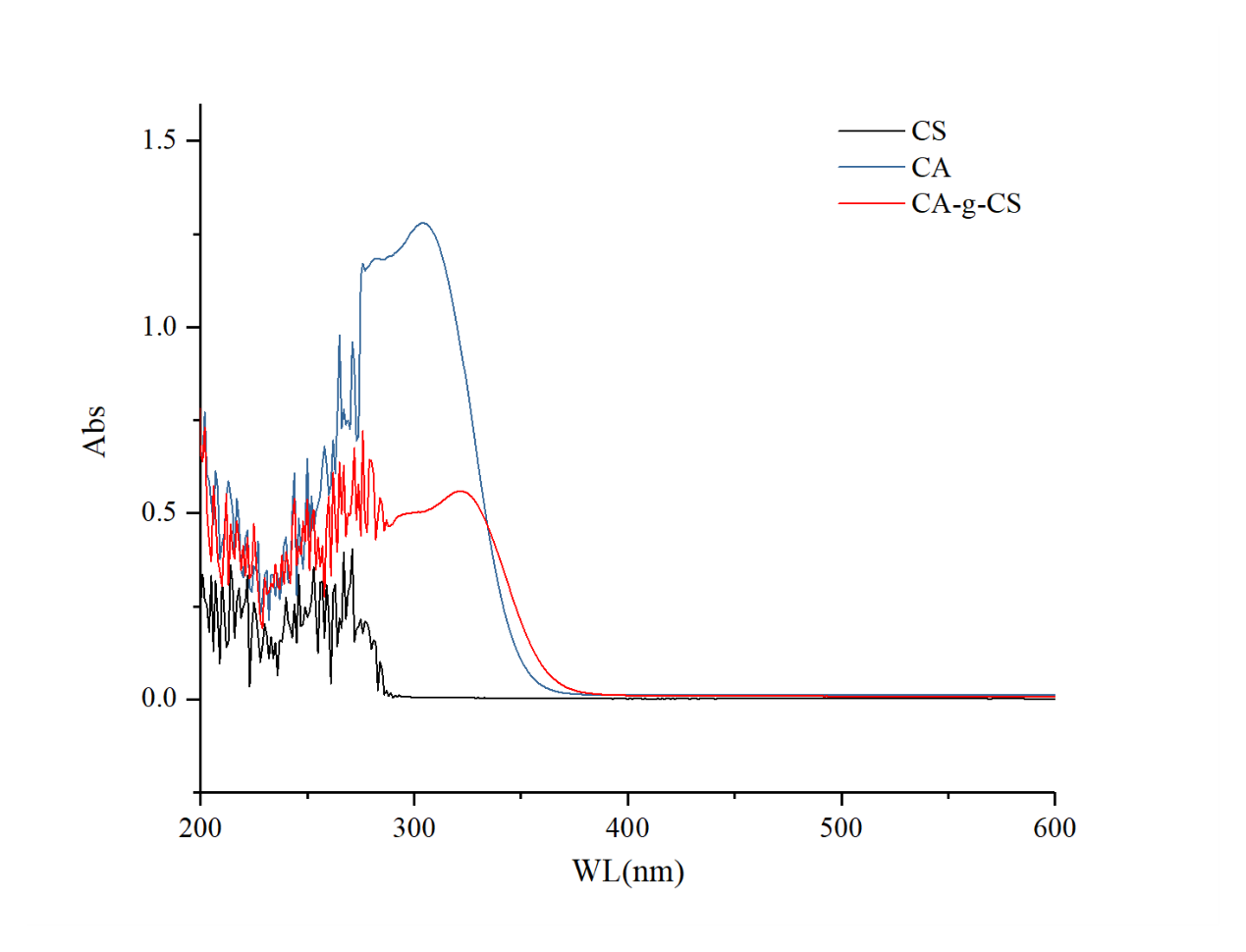


**Supplementary FIGURE 1**

CS, CA, CA-g-CS UV-Vis spectra.

**2. CS, CA, CA-g-CS Fourier transform infrared spectra.**

Take about 2mg of sample, take about 100mg of potassium bromide, grind them well and press them together to 20MPa to compress the tablets, and then directly perform the IR test in the wave number range of 400-4000cm^-1^. The resolution of the spectrometer was 4cm^-1^, and the signal-to-mania ratio was 50,000:1, with 32 scans. The FT-IR spectra of CS, CA, and CA-g-CS are shown in Figure 2. In the FT-IR spectra of CS, the stretching vibrations at 3433.84 and 2874.10 cm^-1^ are attributed to the -OH and C-H groups, respectively, while the C -The absorption peaks at 1422.93 and 1383.35 cm^-1^ corresponded to the bending vibration of -CH_2_ and the symmetric deformation of -CH_3_, respectively. the new absorption peak of CA-g-CS at 1619.55 cm^-1^ was probably caused by the stretching vibration of caffeic acid C=C, which proved that caffeic acid could be successfully grafted onto chitosan.

**Supplementary FIGURE 2**

CS, CA, CA-g-CS Fourier transform infrared spectra.


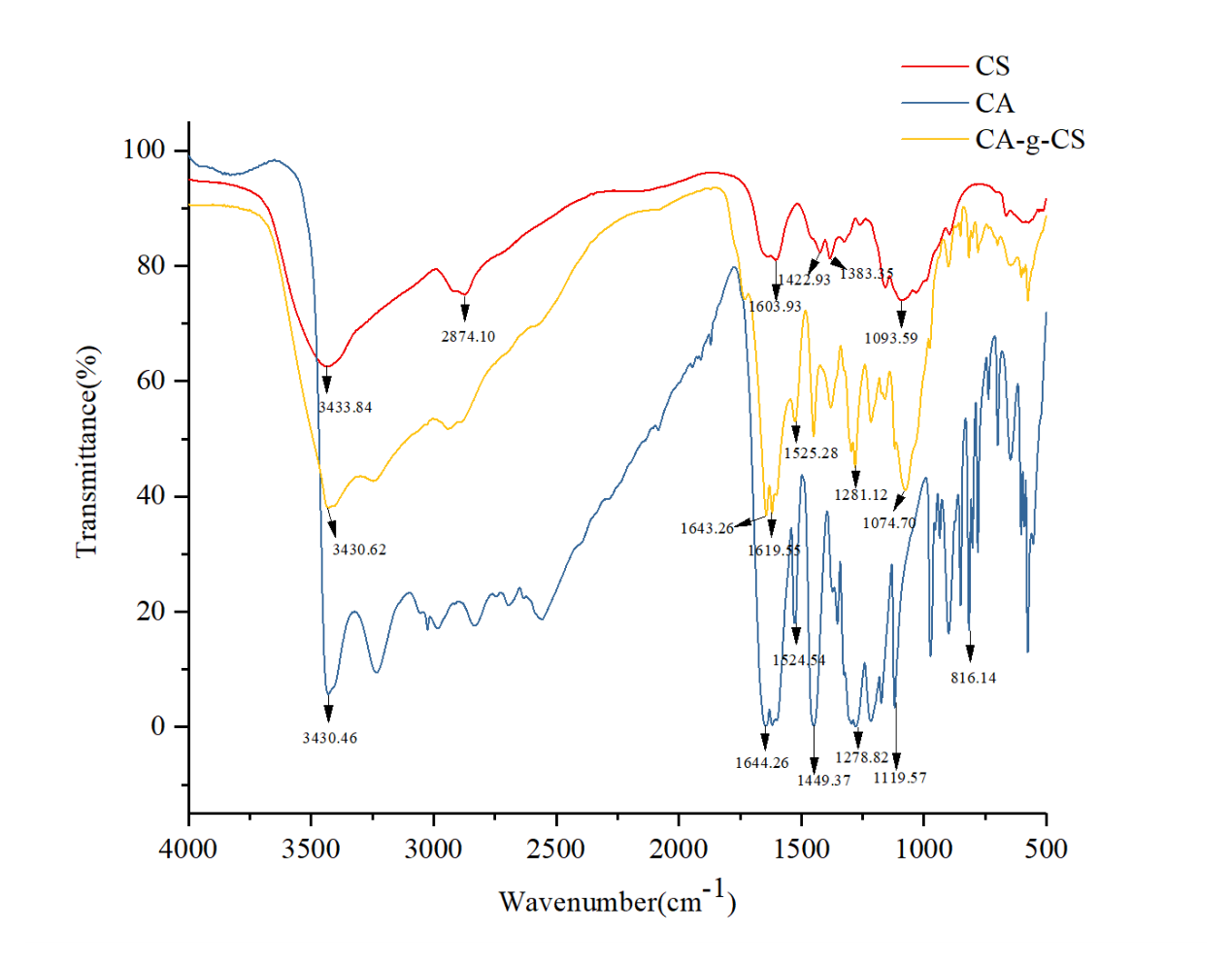

Supplement: Supplementary file 1 [file Data_Sheet_1.docx]
